# Alpha 2-antiplasmin deficiency in a Sudanese child: a case report

**DOI:** 10.1186/s13256-021-02813-6

**Published:** 2021-05-07

**Authors:** Bashir Abdrhman Bashir Mohammed

**Affiliations:** Hematology Department, Faculty of Medical Laboratory Sciences, Port Sudan Ahlia College, Port Sudan, Sudan

**Keywords:** Alpha 2-antiplasmin (α_2_-AP), Fibrinolysis, Bleeding tendency, Sudan

## Abstract

**Background:**

The plasma serine protease inhibitor alpha 2-antiplasmin (α_2_-AP, otherwise known as α_2_-plasmin inhibitor) is a rapid-acting plasmin inhibitor recently found in human plasma, which seems to have a significant role in the regulation of *in vivo* fibrinolysis. Congenital deficiency of α_2_-AP is extremely uncommon.

**Case presentation:**

We report here a case of absolute deficiency of α_2_-AP in an 11-year-old Sudanese boy, who had a lifelong intermittent hemorrhagic tendency (gum bleeding, epistaxis, and exaggerated bleeding after trauma). Coagulation tests including prothrombin time, partial thromboplastin time, thrombin time, bleeding time, platelet count, clot retraction test, antithrombin, and factor VIII levels were within normal limits. Hepatic function tests and complete blood count were also normal. The main interesting finding in this patient was that the whole blood clot lysis was extremely fast, completed within 5–8 hours. The second abnormal finding is that the euglobulin clot lysis time was short. Nevertheless, the concentration of α_2_-AP in the patient's plasma was 0.2 IU/ml (reference range is 0.80–1.20 IU/ml). The addition of pooled plasma (with normal α_2_-AP) to the patient's whole blood corrected the accelerated fibrinolysis.

**Conclusion:**

The study showed that α_2_-AP deficiency resulted in uninhibited fibrinolysis that caused the hemorrhagic tendency in this patient. Thus, this report demonstrates the significant role of α_2_-AP in coagulation.

## Background

**Fig. 1:**
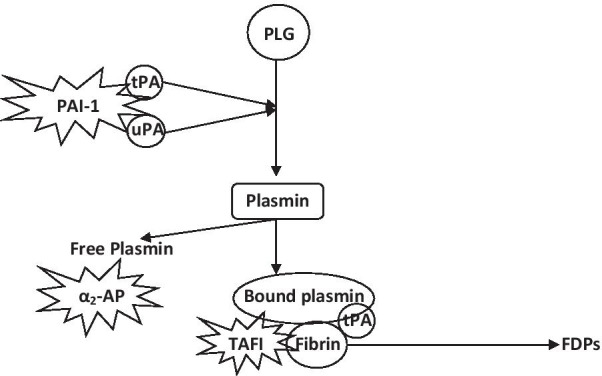
Fibrinolysis: role of alpha 2-antiplasmin. *PLG* plasminogen, *tPA* tissue plasminogen activator, *uPA* urokinase type plasminogen activator, *PAI-1* plasminogen activator inhibitor-1, *α*_*2*_*-AP* alpha 2-antiplasmin, *TAFI* thrombin-activatable fibrinolysis inhibitor

The alpha 2plasmin inhibitor (previously known as α_2_-antiplasmin [α_2_-AP]) is a glycoprotein with an estimated molecular mass of approximately 51 kDa that works as an essential, rapid-acting physiological inhibitor of free plasmin, and therefore has a basic role in the regulation of fibrinolysis [1]. α_2_-AP is synthesized in the liver. At the same time, it is found in the α-granules of platelets, and secreted when platelets are activated. More recently, α_2_-AP has been discovered in the kidney and brain [2]. α_2_-AP is also a member of the serpin family of proteins. The gene coding for α_2_-AP is located on chromosome 17. The mean value of α_2_-AP in the plasma of healthy subjects is 0.8–1.2 IU/ml, and it inhibits the plasmin generated when all the plasminogen in the plasma is entirely activated to plasmin [3]. The concentration is likewise decreased in liver disease or as a result of its utilization in a fibrinolytic state such as disseminated intravascular coagulation (DIC), amyloidosis, solid tumor, acute promyelocytic leukemia (APL), or thrombolytic treatment [4]. Two types of α_2_-AP are available in the blood: 70% of α_2_-AP binds plasminogen and has inhibitory activity, while the remaining 30% is in a nonbinding form. The nonbinding form is the result of degradation of the binding form and has minimal inhibitory action [5]. Fibrinolysis is managed by α_2_-AP in three different ways: first, by the development of a stoichiometric complex with plasmin, where α_2_-AP is significantly more effective in inhibiting free plasmin than plasmin bound to the fibrin clot, and this permits limited plasmin production on the fibrin clot (Fig. 1); second, by the inhibition of plasmin adsorption on the fibrin thrombus, where α_2_-AP is covalently bound into the fibrin clot by factor XIIIa, inhibiting fibrinolysis by fibrin coagulation; and third, by preventing the engagement of plasminogen in the fibrin clot. α_2_-AP responds quickly with plasmin to form a steady plasmin-antiplasmin complex. This communication is key to the physiological control of fibrinolysis and irreversibly blocks plasmin action, which in turn partially debases α_2_-AP. The plasmin-antiplasmin complex is cleared more quickly from the circulation [6]. When the amount of plasmin generated exceeds the capacity of α_2_-AP to neutralize plasmin, the α_2_-macroglobulin can act as a less functional reinforcement inhibitor. In this respect, α_2_-AP represents roughly 90% of free plasmin inhibition *in vivo* [4].

In this report, we describe a patient with an inherited deficiency of α_2_-AP. This patient had shown a bleeding tendency since early life. α_2_-AP deficiency, which induced increased fibrinolytic activity, was the only abnormality present. To the best of the author’s knowledge, this is the first case of α_2_-AP deficiency to be reported in Sudan.

## Case presentation

**Fig. 2:**
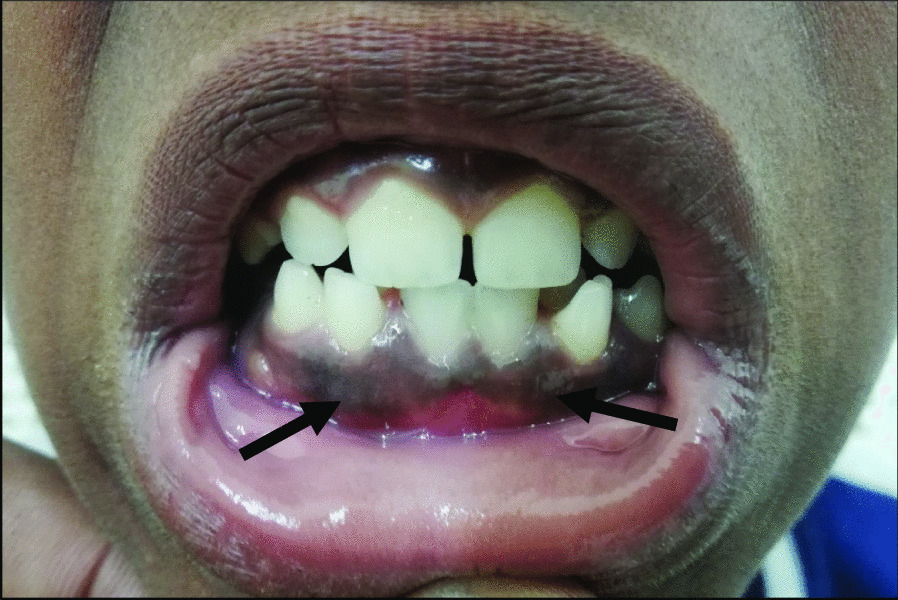
Gingival bleeding in the patient (black arrows)

An 11-year-old Sudanese boy presented with frequent bleeding diathesis (ecchymosis, epistaxis, and gingival bleeds) (Fig. 2). The most commonly encountered type of hemorrhage was extensive bleeding after trauma followed by subcutaneous bleeding. The patient had a history of delayed bleeding episodes since the day of circumcision, which took approximately 1 month to stop. Repeated episodes of epistaxis were also reported. He did not receive blood or blood product transfusion. He reported bleeding in the subcutaneous tissue following trauma when he was playing with his friends. Hemorrhage into joints also occurred after trauma. Spontaneous joint bleeding was not reported. Pain and slight swelling of muscles sometimes persisted for months. Looking into the family history, all family members were normal except his mother. The mother had a history of severe interval bleeding diathesis (menorrhagia). The patient had no known chronic sickness, drug allergies, or any developmental abnormality.

On examination, the patient was neither icteric nor cyanosed, and was not dysmorphic. There was no noticeable change when checking the ear, nose, and throat. The cardiopulmonary examination was normal. The patient’s central nervous system examination showed good sensation and reflexes. He had no skin rash or organomegaly. The liver function tests and complete blood count were all normal. Viral screening for human immunodeficiency virus (HIV), hepatitis B virus (HBV), and hepatitis C virus (HCV) were negative (Table 1).

**Table 1:** Routine investigation of the patient

Variables	Patient results	Normal or control
White blood cells (×10^9^/l)	7.1	4–10
Red blood cells (×10^12^/l)	4.56	3.5–5.5
Hemoglobin (g/dl)	12.5	12–16
Hematocrit (%)	37.3	35–47
Mean corpuscular volume (fl)	81.8	78–98
Mean corpuscular hemoglobin (pg)	27.4	26–35
Mean corpuscular hemoglobin concentration (%)	33.8	30–36
Red cell distribution width-CV (%)	11.7	11.5–14.5
Absolute lymphocyte count (×10^9^/l)	5.8	6–8.3
Absolute neutrophil count (×10^9^/l)	1.3	1.5–6.0
Platelet count (×10^9^/l)	345	150–400
Mean platelet volume (fl)	7.9	7.5–10.4
Platelet distribution width (fl)	9.3	9–17
Platelet-large cell ratio (%)	12.7	13–43
Reticulocyte count (%)	0.6	0.5–3%
Bilirubin total (mg/dl)	0.76	< 1.1
Bilirubin direct (mg/dl)	0.09	< 0.3
Total protein (g/dl)	7.4	6.6–8.3
Albumin (g/dl)	4.2	3.5–5.0
Alanine transaminase (ALT) (U/l)	22	Up to 41
Aspartate transaminase (AST) (U/l)	18	Up to 40
Alkaline phosphatase (ALP) (U/l)	120	< 300
Hepatitis C virus screening (HCV)	Negative	–
Hepatitis B virus screening (HBV)	Negative	–
Human immunodeficiency virus (HIV) screening	Negative	–

*CV* coefficient of variation

The coagulation tests were performed when he was asymptomatic. The main abnormal investigations were as follows: fast lysis of whole blood clots and shortened euglobulin lysis time. The other tests including clotting time, prothrombin time (PT), partial thromboplastin time (PTT), and thrombin time (TT) were all within the normal values. This suggests that there were no abnormal changes in blood coagulation. This was further confirmed by the testing of coagulation factor activity. Platelet count, bleeding time, and von Willebrand factor were also normal (Table 2).

**Table 2:** Hemostatic outcomes for the patient

Variables	Patient results	Normal or control
Bleeding time (minutes)	3.49	2–7
Clotting time (minutes)	7.39	5–15
Clot retraction (%)	60	30–120
Platelet count (µl)	345 × 10^9^	150–450 × 10^9^
Prothrombin time (seconds)	14.7	12–16
Partial thromboplastin time (seconds)	31.6	26–43
Thrombin time (seconds)	16.4	8–18
Antithrombin (%)	88	78–126
d-Dimer (mg)	< 0.1	Up to 0.3
Von Willebrand factor : Ag (U)	1.1	0.15–2.4
F I (mg)	343	200–400
F VIII (%)	61	50–186
F X, %	79	50–150
F XIII (%)	91	60–130
Euglobulin lysis time (minutes)	54	90–240
Whole clot lysis time (hours)	5–8	Negative at 24 hours
α_2_-antiplasmin (IU)	0.2	0.8–1.2

**Fig. 3:**
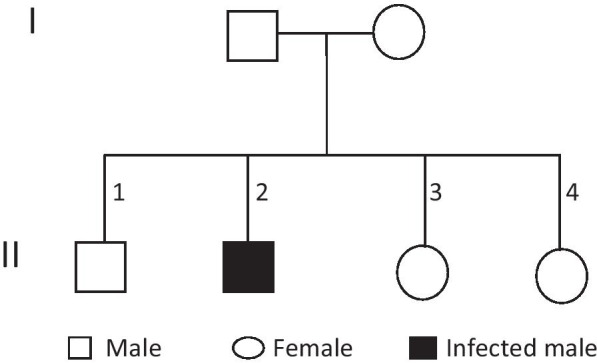
Autosomal recessive trait pedigree of the Sudanese male patient with α_2_-AP deficiency. Each subject can be located in the pedigree by a Roman numeral indicating generation. The patient (II 2) possessing 0.2 IU/ml α_2_-AP in plasma was believed to be a heterozygote

Concerning the clot lysis test, the blood was collected and promptly placed in a glass test tube and incubated at 37°C. The clot was formed and retracted normally, but then underwent lysis. Following a few hours of incubation (5–8 hours with interval assessment), the clot was hardly visible, and it was just a small strand of fibrin. The fibrinogen and d-dimer concentration were within normal limits. To determine the status of fibrinolysis, euglobulin lysis time was calculated, and the result indicated shortened lysis time (Table 2). To further investigate, we treated the patient’s plasma with a pooled normal plasma and repeated the euglobulin lysis time, which returned to normal. However, in such cases, it is preferable to use thromboelastometry to verify the diagnosis. Unfortunately, due to a lack of facilities, this technique was not performed. The defect seems to be inherited as an autosomal recessive trait (Fig. 3).

## Discussion and conclusion

Human plasma contains numerous inhibitors of fibrinolysis. It may maintain a condition of harmony with activating segments of fibrinolysis [5]. An increased propensity for developing thromboembolism has been attributed to decreased levels of natural regulatory anticoagulant proteins [7]. The case depicted in this report indicates that a serious deficiency of α_2_-AP in plasma is associated with hyperfibrinolysis activity and a bleeding tendency. Therefore, this case demonstrates the significant role of α_2_-AP in the coagulation and fibrinolytic system.

In the absence of α_2_-AP, plasmin dissociates the essential hemostatic platelet-fibrin plug. In this way, it interferes with adequate primary hemostasis. Even if the formation of fibrin is healthy, the subsequent accelerated lysis of the formed fibrin plug (fibrinolysis) induces the onset of delayed bleeding. In this report, the patient has exhibited extensive bleeding after circumcision and minor injuries throughout his life. The patient did not develop hemarthrosis, and the symptoms and signs differed from those of hemophilia. In contrast to hemophilia, the patient’s bleeding occurred mostly after trauma, while spontaneous bleeding was extremely uncommon. Unfortunately, due to a lack of awareness and ignorance on the part of the parents, the patient did not receive care until he reached teenage years and faced repeated bleeding episodes.

The striking change presented in this patient was that the spontaneous lysis of the blood clot was formed *in vitro*. Even though α_2_-AP is known to inhibit some activated clotting factors (XIa or thrombin) in coagulation systems [8], there were no atypical changes in the blood clotting tests of this patient. The unusual hyperfibrinolysis activity was attributed exclusively to a decrease in the level of α_2_-AP. The patient's blood was corrected to its normal level after adding pooled (normal) plasma containing a normal α_2_-AP level. This correction test for fibrinolysis was performed without any change that could influence the action of the inhibitors in the patient’s sample.

When the blood clot forms, fibrinolysis will be activated. Eventually, the plasminogen turned to plasmin and bound to fibrin molecules [9]. Fibrinolysis depends solely on the plasmin that binds to fibrin. α_2_-AP diminishes the rate of binding of plasmin to fibrin, and consequently suppresses fibrin clot lysis. When blood lacks α2-AP, as noted in this report, plasma activation of fibrinolysis will occur freely without any blockers. Therefore, α2-macroglobulin does not inhibit free plasma [10]. The formation of the hemostatic plug may be promptly lysed due to the lack of α_2_-AP. Fibrin strands are the major constituents of the clotting plug, and lysis of these strands may make the hemostatic plug extremely brittle. Since the laboratory results of blood clotting tests and platelet count in this patient were all within normal limits, the patient's bleeding diathesis was absolutely related to the inadequacy of α_2_-AP. Furthermore, the resulting disintegration of the hemostatic plug once formed (when the vessels are injured) is also due to α_2_-AP deficiency.

As the bleeding time, platelet count, clot retraction, and von Willebrand factor Ag levels were normal, these findings indicate that platelet functions were also normal. Despite the hyperfibrinolysis activity in this patient, the fibrinogen concentration was within the normal range. This indicates that abnormal fibrinogenolysis does not occur *in vivo*, as in this patient. It is possible that α_2_-macroglobulin, which was present at normal concentrations in this patient, prevents fibrinogenolysis by hindering the binding of plasmin to fibrin. α_2_-macroglobulin may also enable the inactivation of a variety of proteases (plasmin, kallikrein, and thrombin) [8].

On the other hand, the time of the euglobulin lysis test was shortened considerably. It is commonly believed that euglobulin lysis time reflects the overall fibrinolytic activity of plasma [5]. The shortened time of the euglobulin test for this patient, which was confirmed when α_2_-AP was corrected after the addition of pooled normal plasma, was probably not a direct result of excessive plasminogen activator activity. In reality, this was due to the absence of α_2_-AP in the patient’s blood sample. The direct analysis of the α_2_-AP activity upheld this conclusion. The cause of the α_2_-AP deficiency in this patient’s plasma cannot be determined. It could be due to a lack of synthesis of this protein in the hepatocyte or due to the hypercatabolism of α_2_-AP. Therefore, the origin of the α_2_-AP was most probably congenital. The gold-standard method for measurement of hyperfibrinolysis is by thromboelastometry. This was not performed due to lack of resources.

The clinical severity of the disease is assessed by the magnitude and the site of bleeding. Minor bleeding can be treated by oral antifibrinolytic agents, but more progressive bleeding may demand temporary plasma supplementation. Bleeding at critical locations may likewise require surgical intervention. Patients with congenital or acquired α_2_-AP deficiency who have extreme bleeding should receive fresh-frozen plasma (FFP) transfusion as a source of α_2_-AP. Antifibrinolytic agents such as aminocaproic acid or tranexamic acid, which prevent the production of plasmin and block its action, are used in response to active hemorrhage or as prophylaxis before any dental or surgical procedure [5]. The patient received doses of tranexamic acid and vitamin K that improved his condition.

Although the prevalence of α_2_-AP deficiency as a cause of hemorrhage is quite low as reported in the literature, this report confirms the presence of this disease in Sudan. Deficiency of α_2_-AP could contribute to a high level of free plasmin that can lead to a state of hyperfibrinolysis and bleeding.

## Data Availability

The data sets used and/or analyzed during the current study are available from the corresponding author on reasonable request.

## Abbreviations

α_2_-AP: Alpha 2-antiplasmin
PT: Prothrombin time
PTT: Partial thromboplastin time
TT: Thrombin time
DIC: Disseminated intravascular coagulation
APL: Acute promyelocytic leukemia
HIV: Human immunodeficiency virus
HBV: Hepatitis B virus
HCV: Hepatitis C virus
AST: Aspartate aminotransferase
ALT: Alanine aminotransferase
ALP: Alkaline phosphatase
